# Supplementary material for: scPower accelerates and optimizes the design of multi-sample single cell transcriptomic studies
Source: Nat Commun. 2021 Nov 16;12:6625. doi: 10.1038/s41467-021-26779-7 (PMC8595682; doi:10.1038/s41467-021-26779-7)
Supplement: Supplementary file 1 — Supplementary Information [file 41467_2021_26779_MOESM1_ESM.pdf]

scPower accelerates and optimizes the design of multi-sample single cell transcriptomic studies

Katharina T. Schmid, Barbara Höllbacher, Cristiana Cruceanu, Anika Böttcher, Heiko Lickert, Elisabeth B. Binder, Fabian J. Theis, Matthias Heinig

Supplementary material

Supplementary Figures

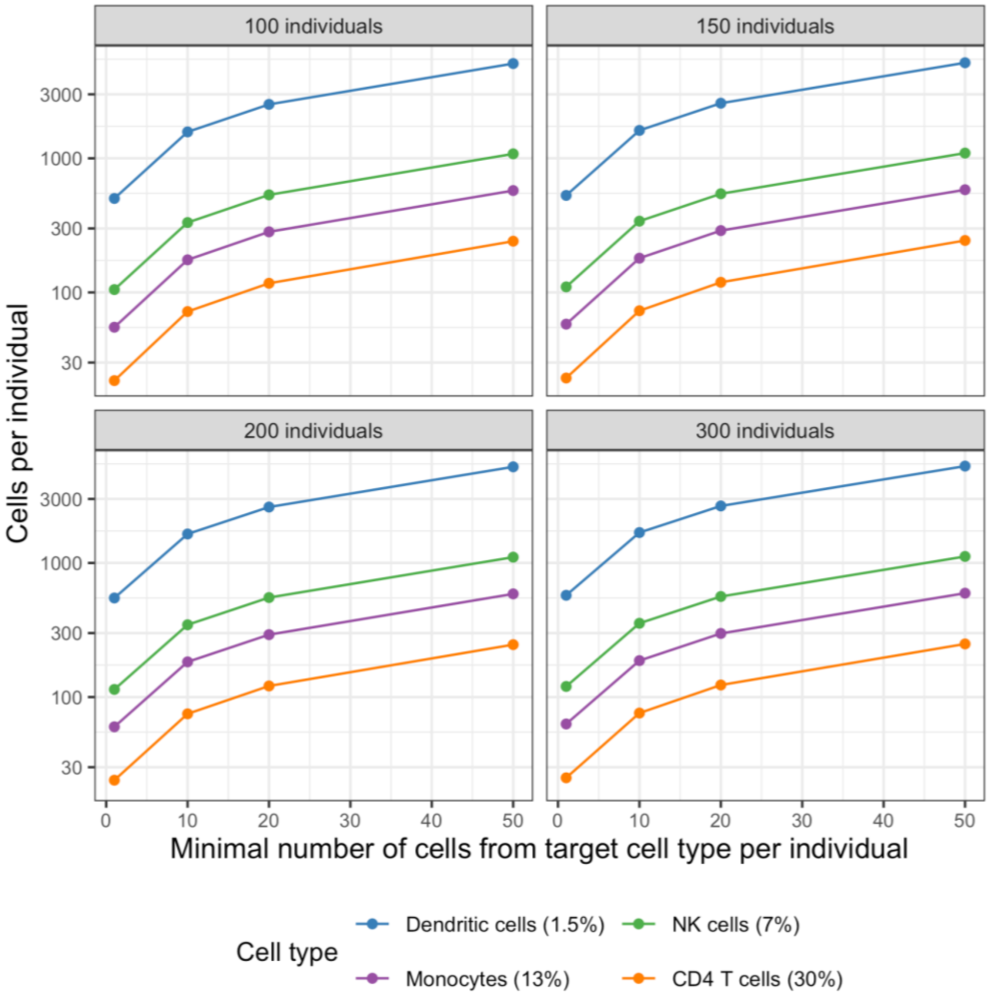

Figure S1: *Power to detect rare cell types*. Required number of cells per individual (y-axis, log scale) to detect the minimal number of cells from a target cell type per individual (x-axis) with a probability of 95%. The probability depends on the total number of individuals and the frequency of the target cell type (purple, red, yellow, green). Note that the required number of cells per sample only counts “correctly measured” cells (no doublets etc), so the number is a lower bound for the required cells to be sequenced.

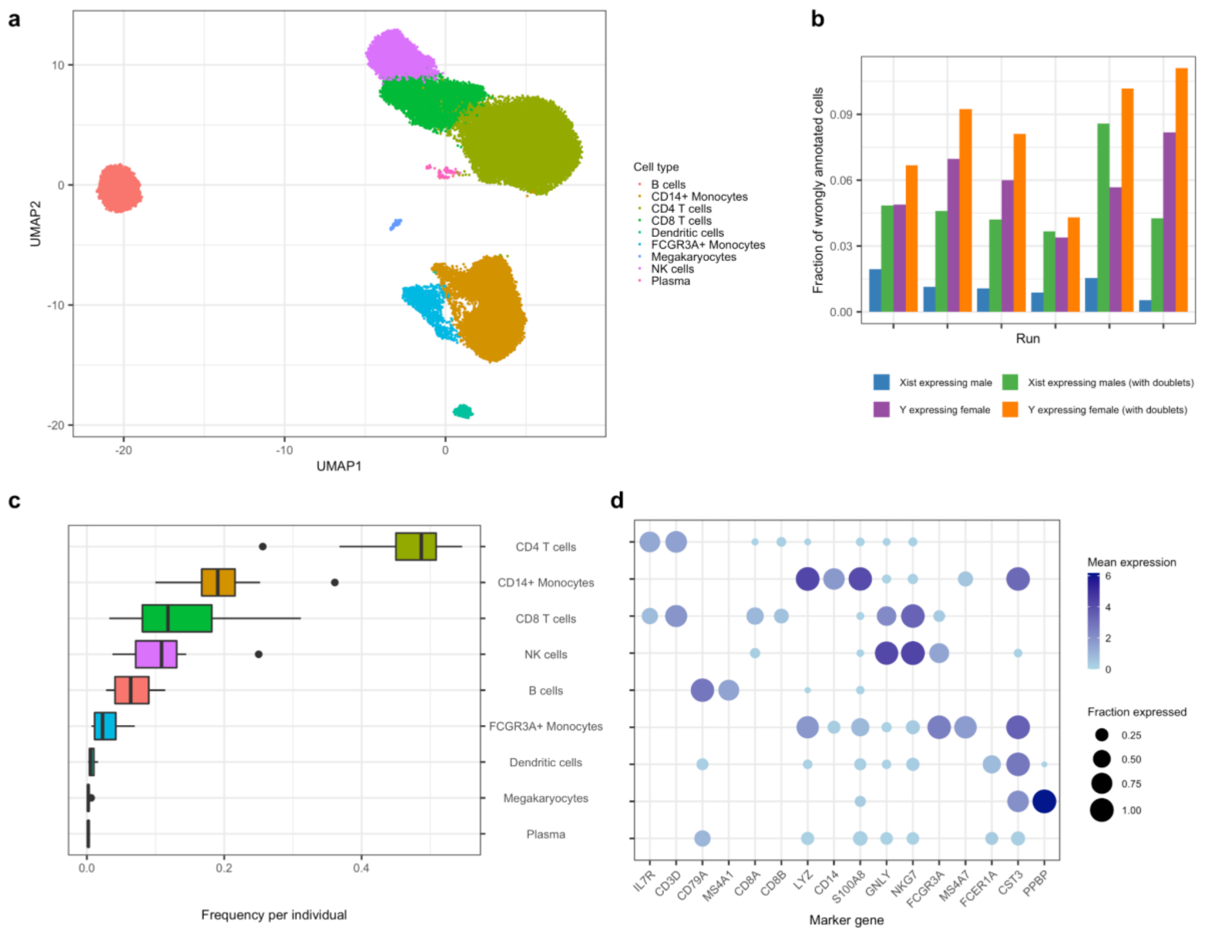

Figure S2: *Training PBMC data set*.

a UMAP visualization of all 6 runs, clustered using Louvain and annotated to cell types using marker genes.

b Evaluation of Demuxlet assignment to the different individuals by testing the expression of sex specific genes per run. The error rate "Xist expressing male" shows which fraction of cells is assigned to a male donor from all cells expressing Xist. The "Y expressing female" shows which fraction of cells is assigned to a female donor from all cells having more reads mapped to chromosome Y than the median value. Both error rates decrease when Demuxlet and Scrublet doublets are removed.

c Cell type frequencies for each individual (n=14 biologically independent samples). Boxplots show medians (centre lines), first and third quartiles (lower and upper box limits, respectively), 1.5-fold interquartile ranges (whisker extents) and outliers (black circles).

d Marker gene distribution over the Louvain clusters. The color of the point visualizes its mean expression in the cluster, the size of the dot in how many cells of the cluster it is expressed (expression level larger than 0).

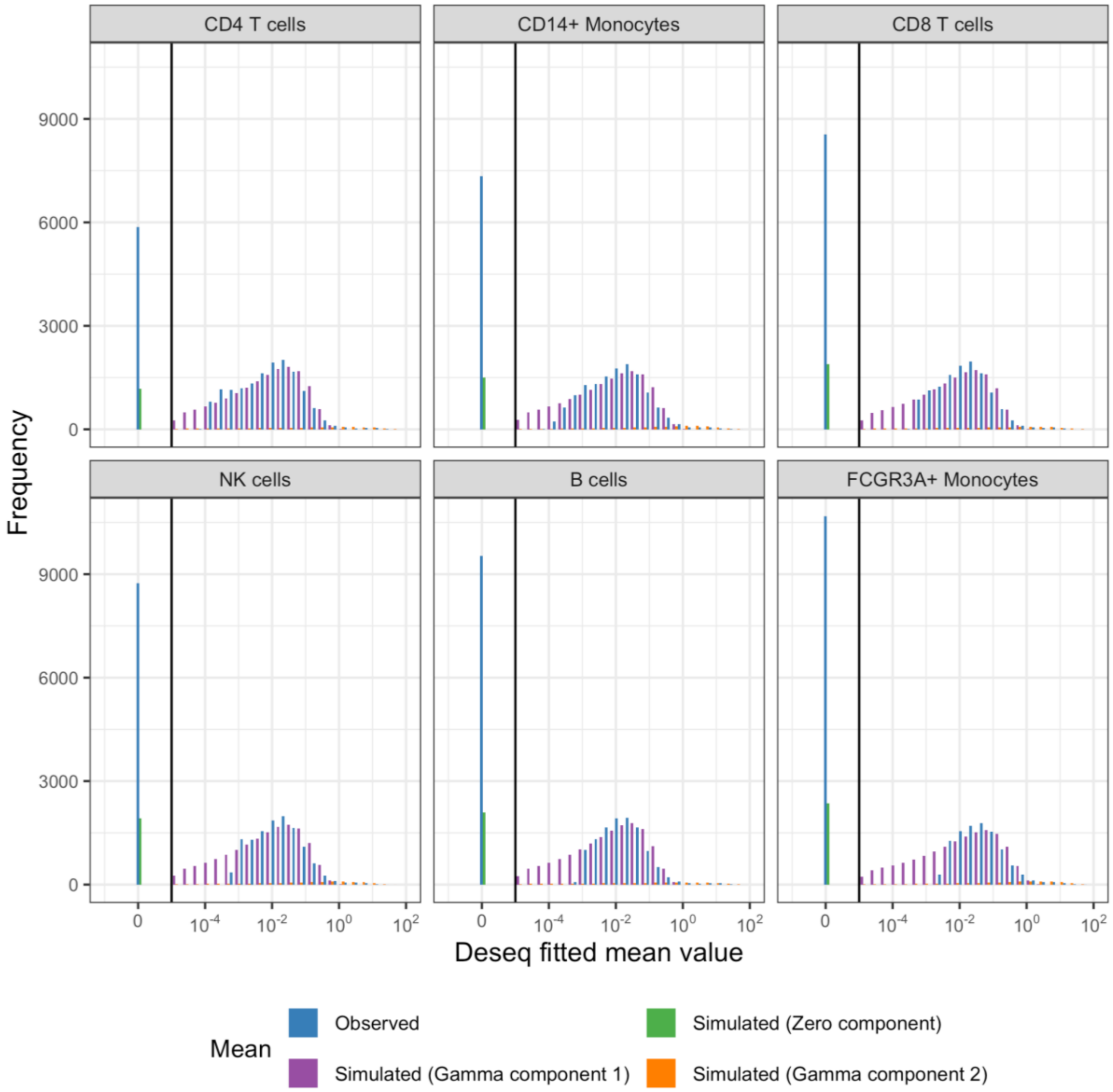

Figure S3: *Evaluation of gamma mixture fits for expression means*. Gamma mixture fit (components in violett, green, orange) over all gene expression means for one batch of the training PBMC data set (**Supplementary Table S2**) compared to the observed distribution (blue). Fitted separately for each cell type (see panel titles), showing the 21,000 highest expressed genes for each cell type.

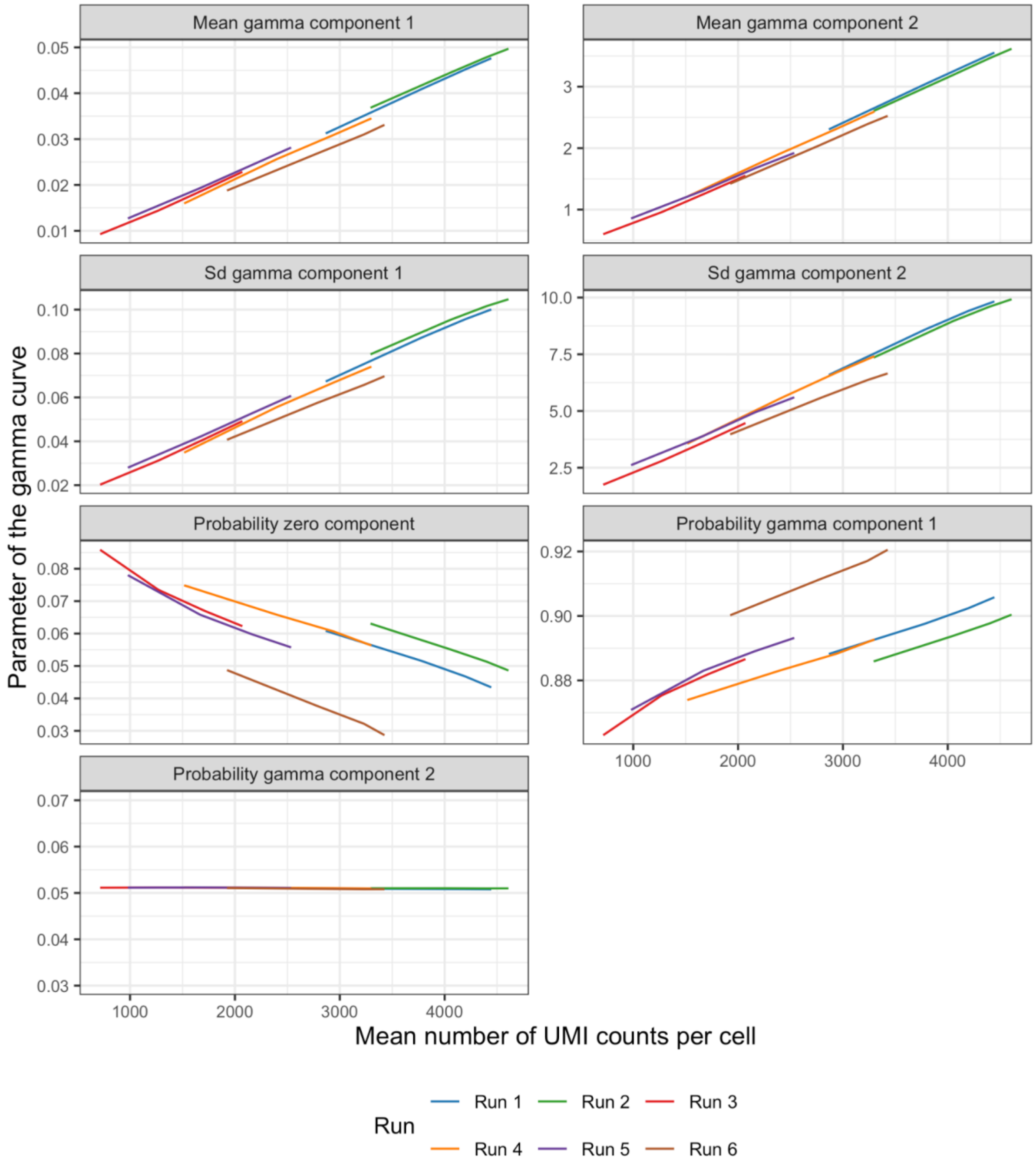

Figure S4: *Relationship between the parameters of the mixture fit and the mean number of UMI counts per cell.* The two left censored gamma components of the mixture distribution are parametrized over their means and standard deviations (row 1 and 2), additionally there are three probability parameters (row 3 and 4) showing the proportion of each of the three components, the zero component and the two gamma components. The fits were performed for each cell type separately, here shown for the CD4 T cells. There is a linear relationship between the mean and standard deviation parameters of the gamma components and the mean UMI counts (row 1 and 2). The probabilities of the zero component and the first gamma component show a linear relationship to the mean UMI counts (row 3). The probability parameter of the second gamma component stays constant (row 4). The other cell types show the same pattern.

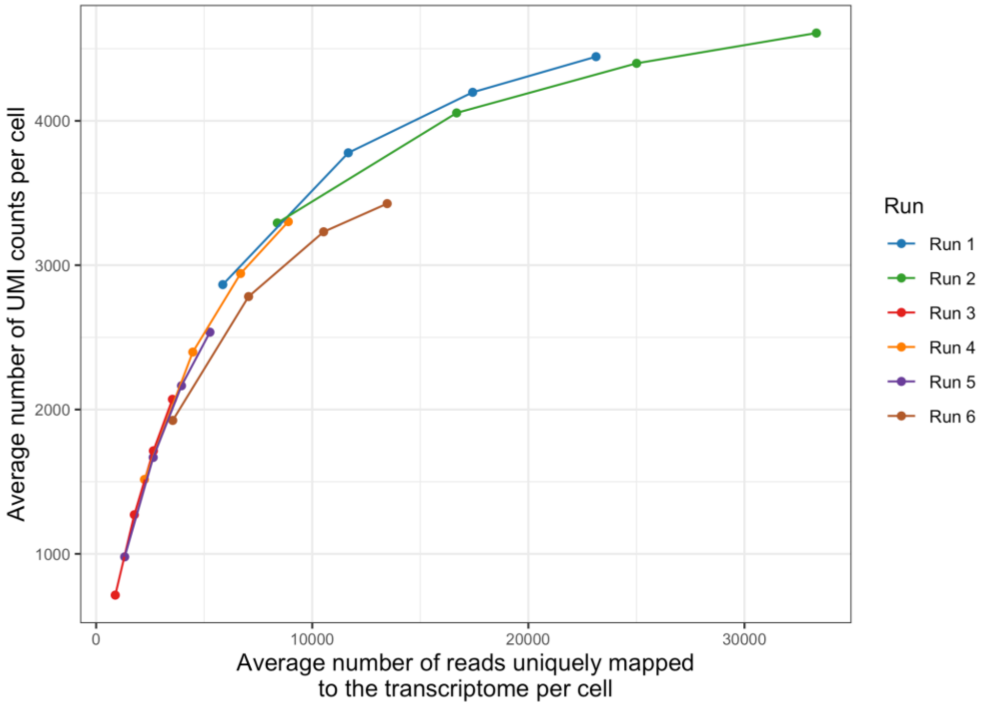

Figure S5: *Relationship between UMI counts per cell and average number of reads that were uniquely mapped to the transcriptome per cell.*

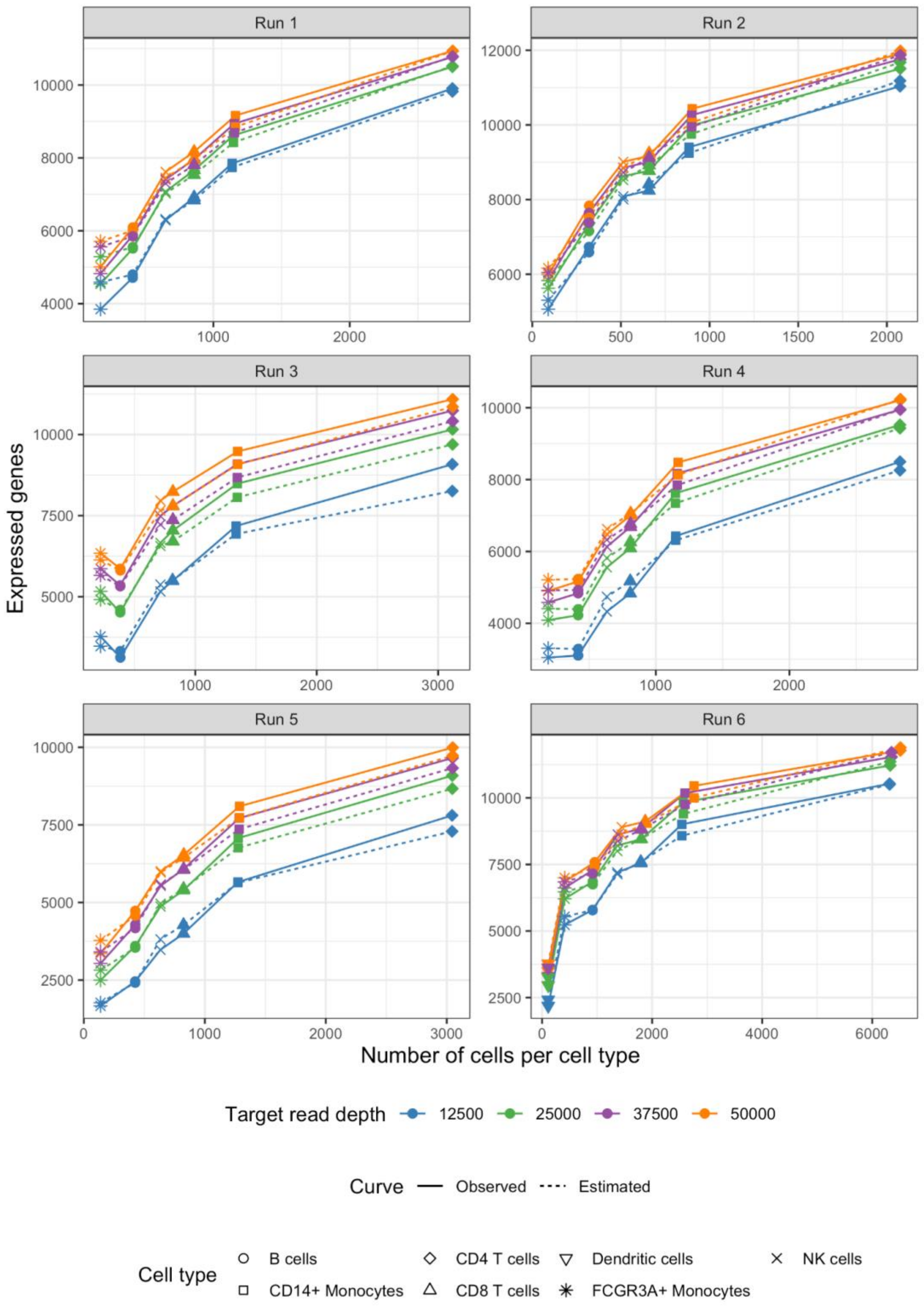

Figure S6: *Expression probability model for a count threshold > 0*. The number of expressed genes expected under our model (dashed line) closely matches the observed number of expressed genes (solid line) dependent on the number of cells per cell type (cell type indicated by point symbol) for all 6 batches (see panel titles) of the training PBMC data set (**Supplementary Table S2**). A gene is defined as expressed if it has > 0 counts in more than 50% of the individuals.

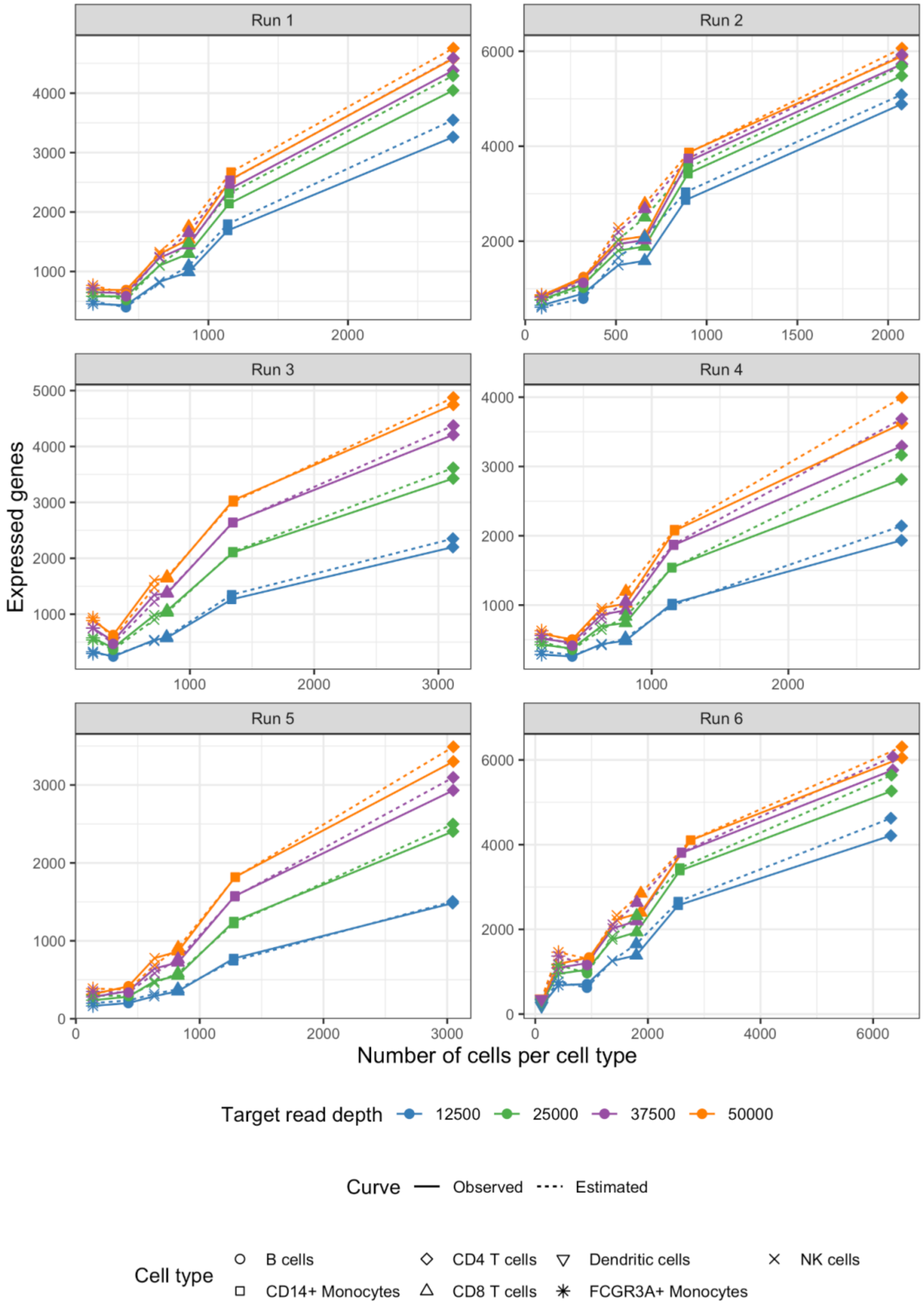

Figure S7: *Expression probability model for a count threshold > 10.* Variant of Figure S6 with a count threshold of 10 instead of 0.

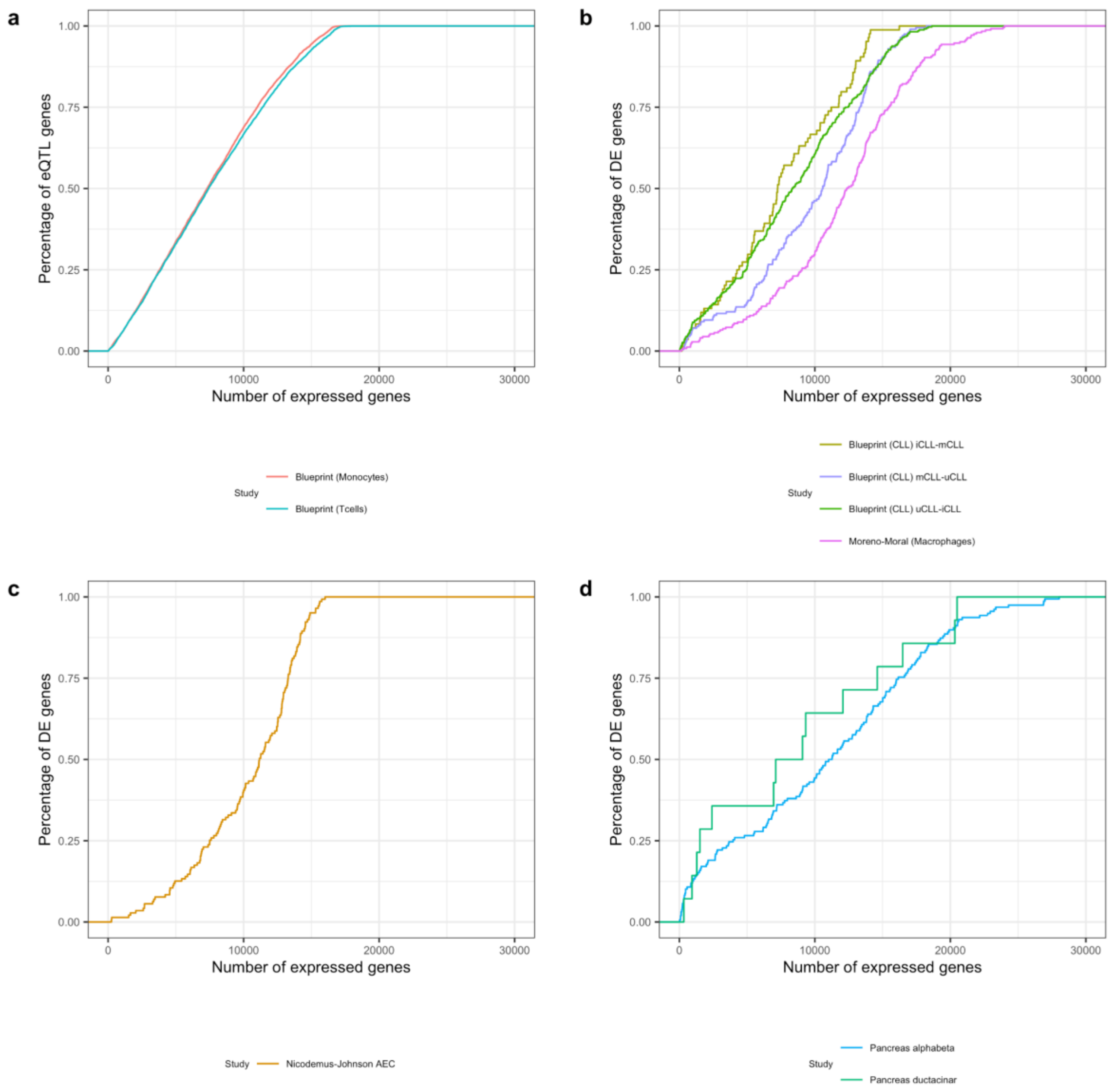

Figure S8: *Expression rank priors from cell type sorted bulk studies*. Gene expression level of DEGs / eQTL genes (y axis) relative to all genes (x axis) (i.e. gene expression ranks) gained from cell type sorted bulk studies for eQTL studies of PBMCs **(a)** and for DE studies of PBMCs **(b)**, lung tissue **(c)** and pancreas tissue **(d)**.

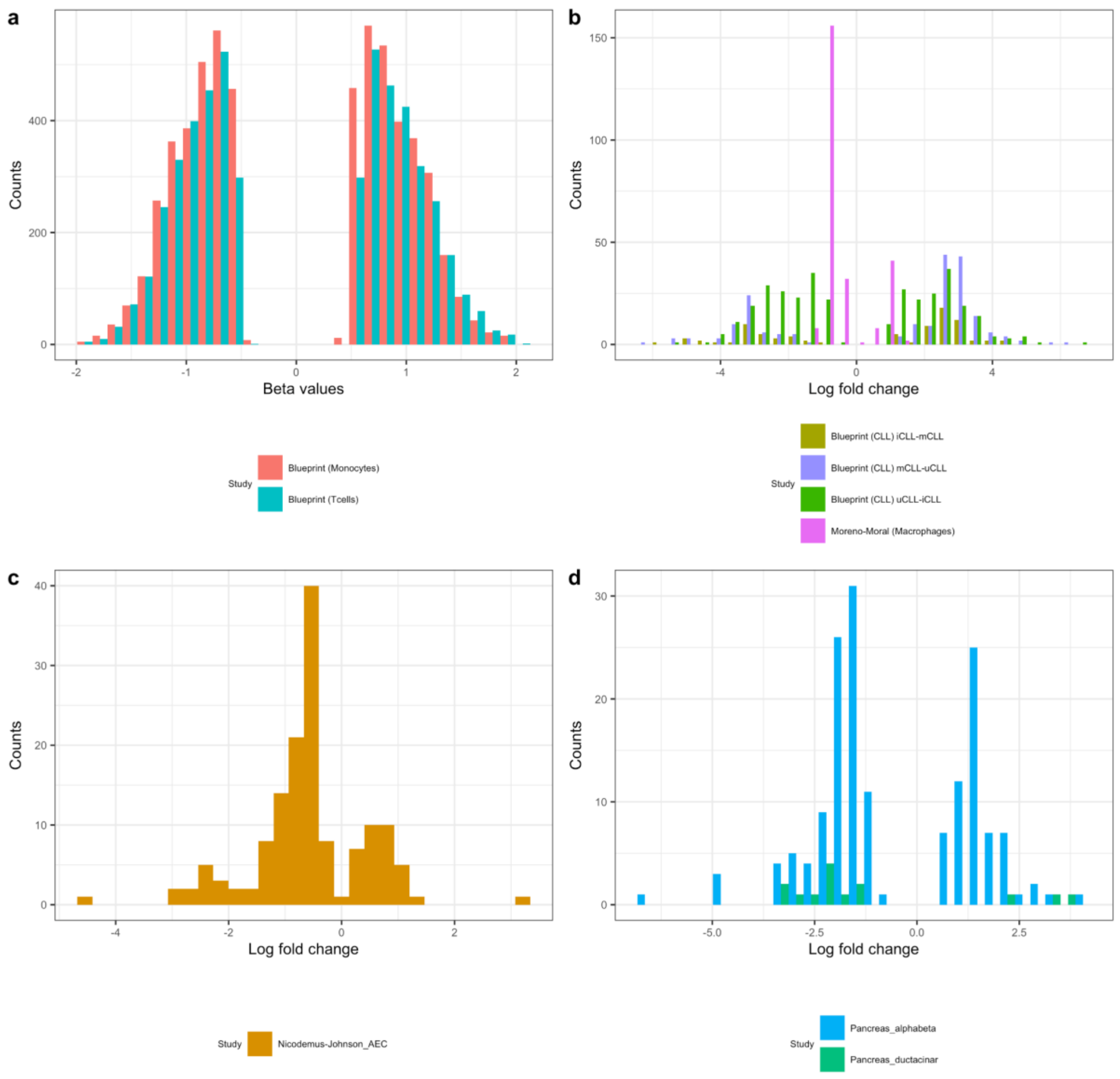

Figure S9: *Effect size priors from cell type sorted bulk studies*. Effect sizes gained from cell type sorted bulk studies for eQTL studies of PBMCs **(a)** and for DE studies of PBMCs **(b)**, lung tissue **(c)** and pancreas tissue **(d)**. The effect size is quantified as beta values for eQTL studies and as log fold changes for DE studies.

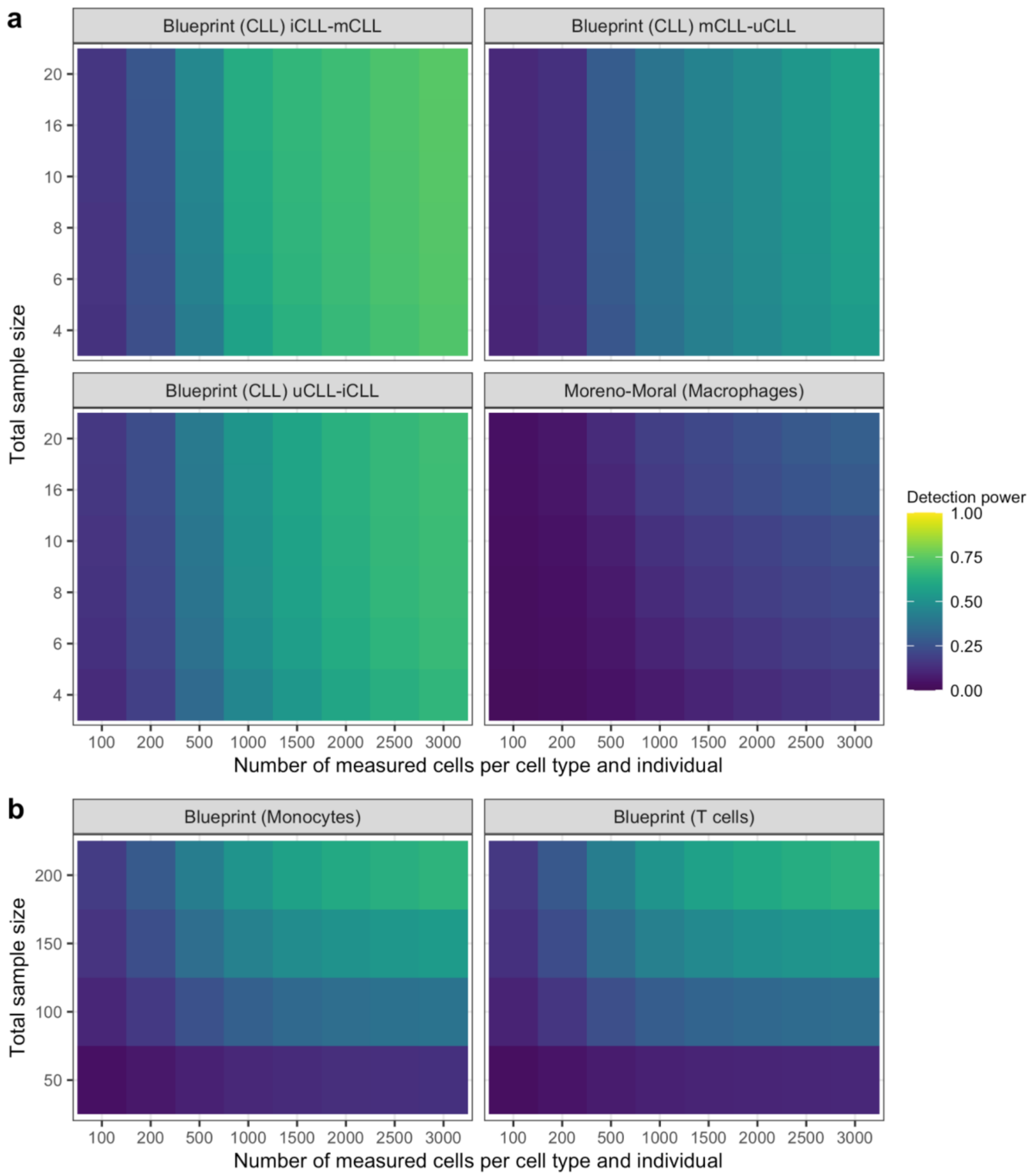

Figure S10: *Detection power using observed priors from reference studies*. Detection power for DE genes **(a)** and eQTL genes **(b)** dependent on the study, total sample size and the number of measured cells per cell type for a transcriptome mapped read depth per cell of 20,000. The detection power is the product of the probability that the gene is expressed and the power to detect it as a DE or eQTL gene, respectively, assuming that it is expressed. The fold change for DE genes and the R^2 for eQTL genes is taken from published studies, together with the expression rank of the genes (studies shown in panel titles). The expression profile and expression probabilities in a single cell experiment with a specific number of samples and measured cells was estimated using our expression prior, setting the definition for expressed to > 10 counts in more than 50% of the individuals. Multiple testing correction was performed by using FDR adjusted p-values for DE analysis and FWER adjusted p-values for eQTL analysis.

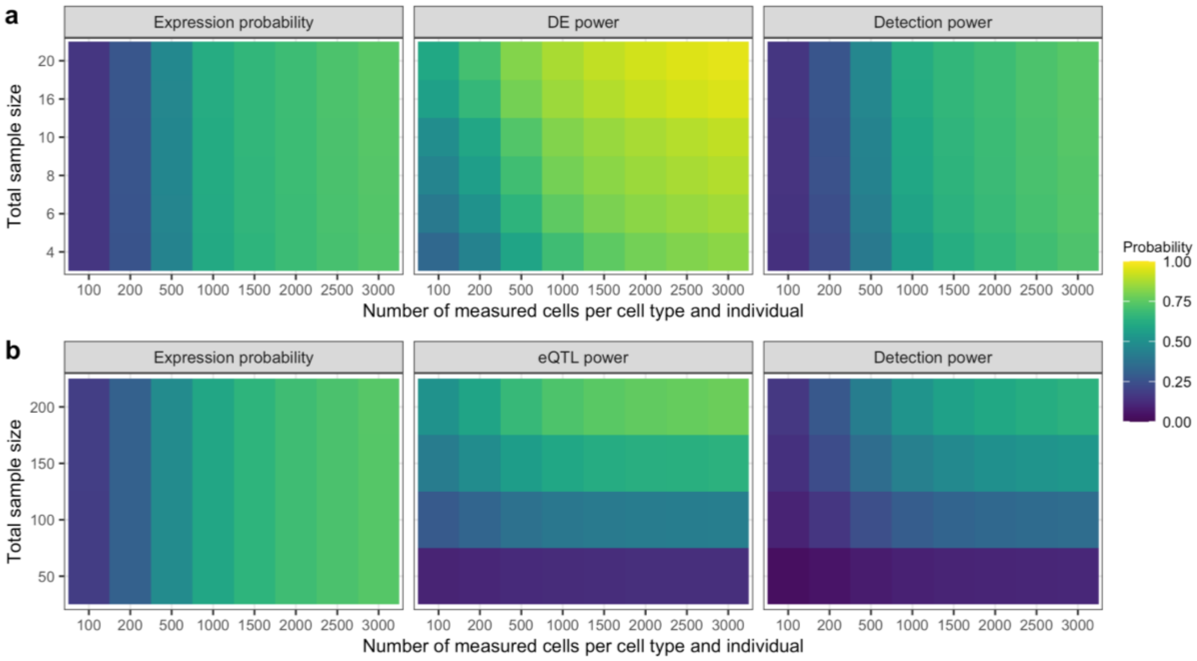

Figure S11: *Variant of Main Figure 3a,b created with FWER for both DE and eQTL analysis - Expression probability, DE/eQTL power and overall detection power.* In contrast to Main Figure 3a,b, FWER adjusted p-values were also used for DE power. Power estimation using data driven priors for DE genes **(a)** and eQTL genes **(b)** dependent on the total sample size and the number of measured cells per cell type. The fold change for DEGs and the R^2 for eQTL genes were taken from published studies, together with the expression rank of the genes. For **(a)**, the Blueprint CLL study with comparison iCLL vs mCLL was used, for **(b)**, the Blueprint T cell study. A gene is defined as expressed with > 10 counts in more than 50% of the individuals.

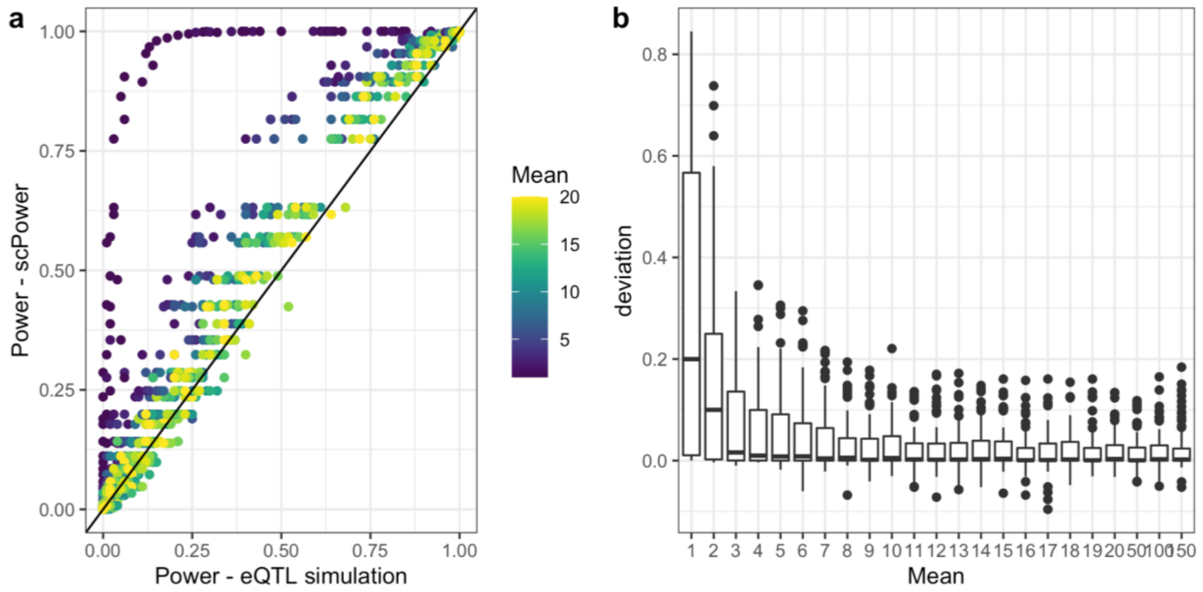

Figure S12: *Relation between eQTL power and expression mean in a simulation study.*

a The simulated eQTL power is compared to the analytic power calculated with scPower for a range of effect sizes (R^2 between 0.1 and 0.6), sample sizes (between 20 and 150) and Bonferroni-corrected p-values (between 0.05/(10*1,000) and 0.05/(10*10,000)). The color coding shows the mean count value.

b The deviation between the analytic power and the simulated power is stratified by the expression mean used in the simulation. Boxplots show medians (centre lines), first and third quartiles (lower and upper box limits, respectively), 1.5-fold interquartile ranges (whisker extents) and outliers (black circles).

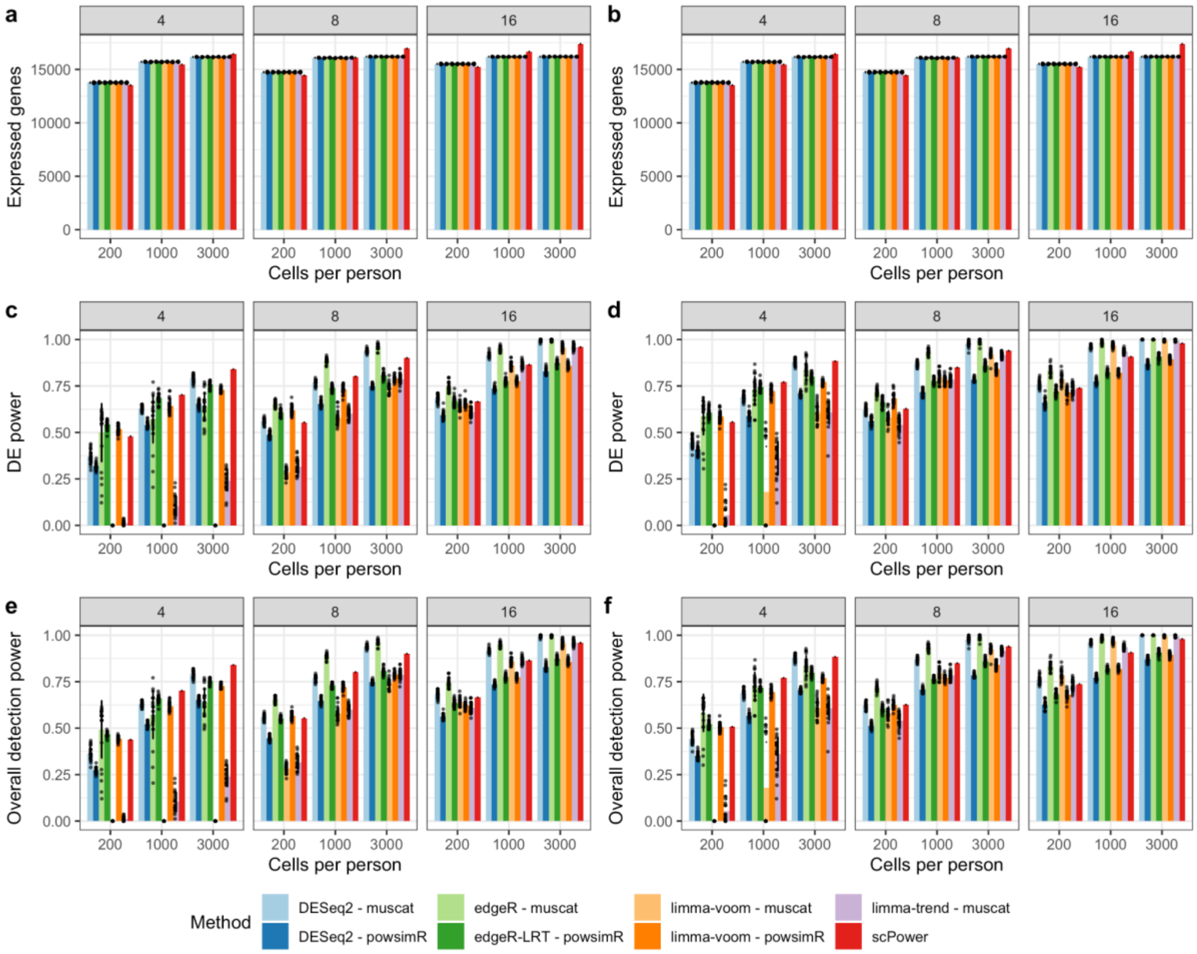

Figure S13: *Comparison of scPower with the simulation-based methods powsimR and muscat in combination with different DE methods.* Difference in number of expressed genes **(a,b)**, DE power **(c,d)** and overall detection power **(e,f)** between scPower and simulations. The adapted version of powsimR (see methods) was run with DESeq2, edgeR-LRT and limma-voom, using the mean-ratio method (MR) for normalization. The adapted version of muscat (see methods) was run with DESeq2, edgeR, limma-trend and limma-voom. The power was evaluated for sample sizes of 4, 8 and 16 (see panel titles) and for 200, 1000 and 3000 cells per person (x axis). Both FWER adjusted power **(a,c,e)** and FDR adjusted power **(b,d,f)** were evaluated. The barplots represent the mean power over n=25 simulation runs of powsimR and muscat, the error bar shows the standard deviation and the points represent each individual simulation run. scPower as an analytic solution provides always the same result (so n=1 here).

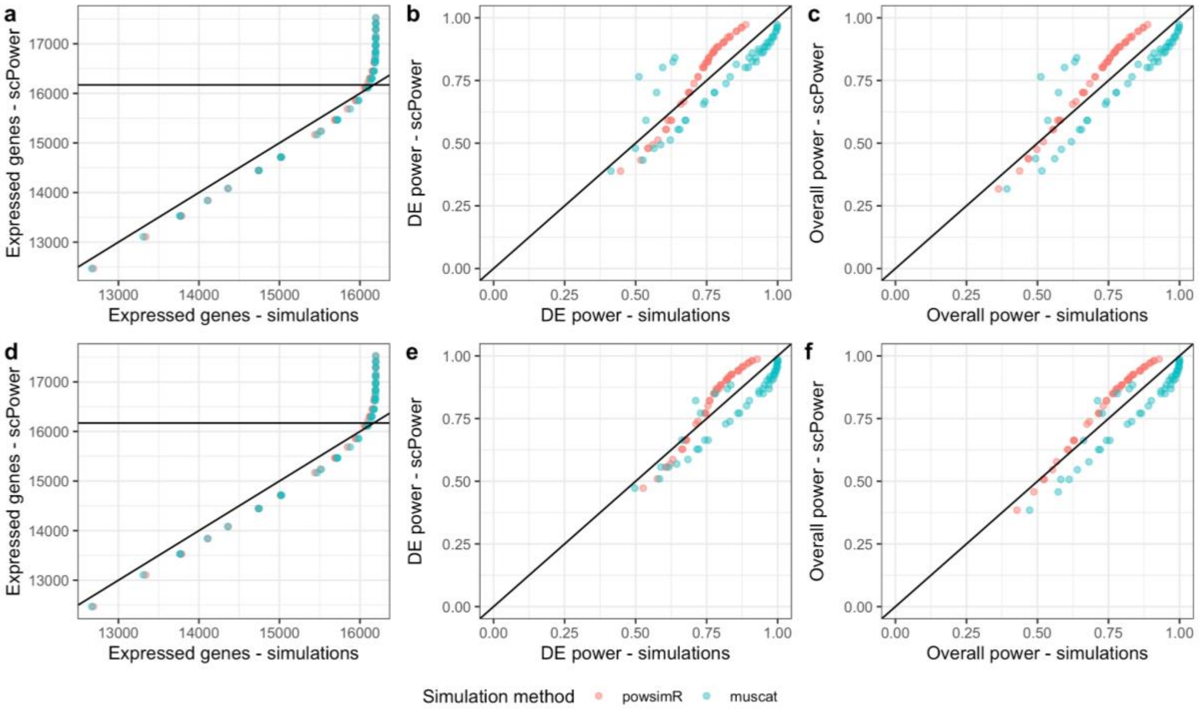

Figure S14: *Comparison between scPower with the simulation-based methods powsimR and muscat over a large range of experimental design.* Difference in number of expressed genes **(a,d)**, DE power **(b,e)** and overall detection power **(c,f)** between scPower (y axis) and simulations of powsimR and muscat (x axis). The adapted version of powsimR (see Methods) was run with edgeR-LRT using the mean-ratio method (MR) for normalization and the adapted version of muscat with edgeR. The power was evaluated for all combinations of main Figure 3 (sample sizes of 4, 6, 8, 16 and 20 and cells per person of 100, 200, 500, 1000, 1500, 2000, 2500 and 3000). Both FWER adjusted p-values **(a-c)** and FDR adjusted p-values **(d-f)** were evaluated. In **(a)** and **(d)**, the horizontal line represents the maximum number of possible simulated genes for powsimR .

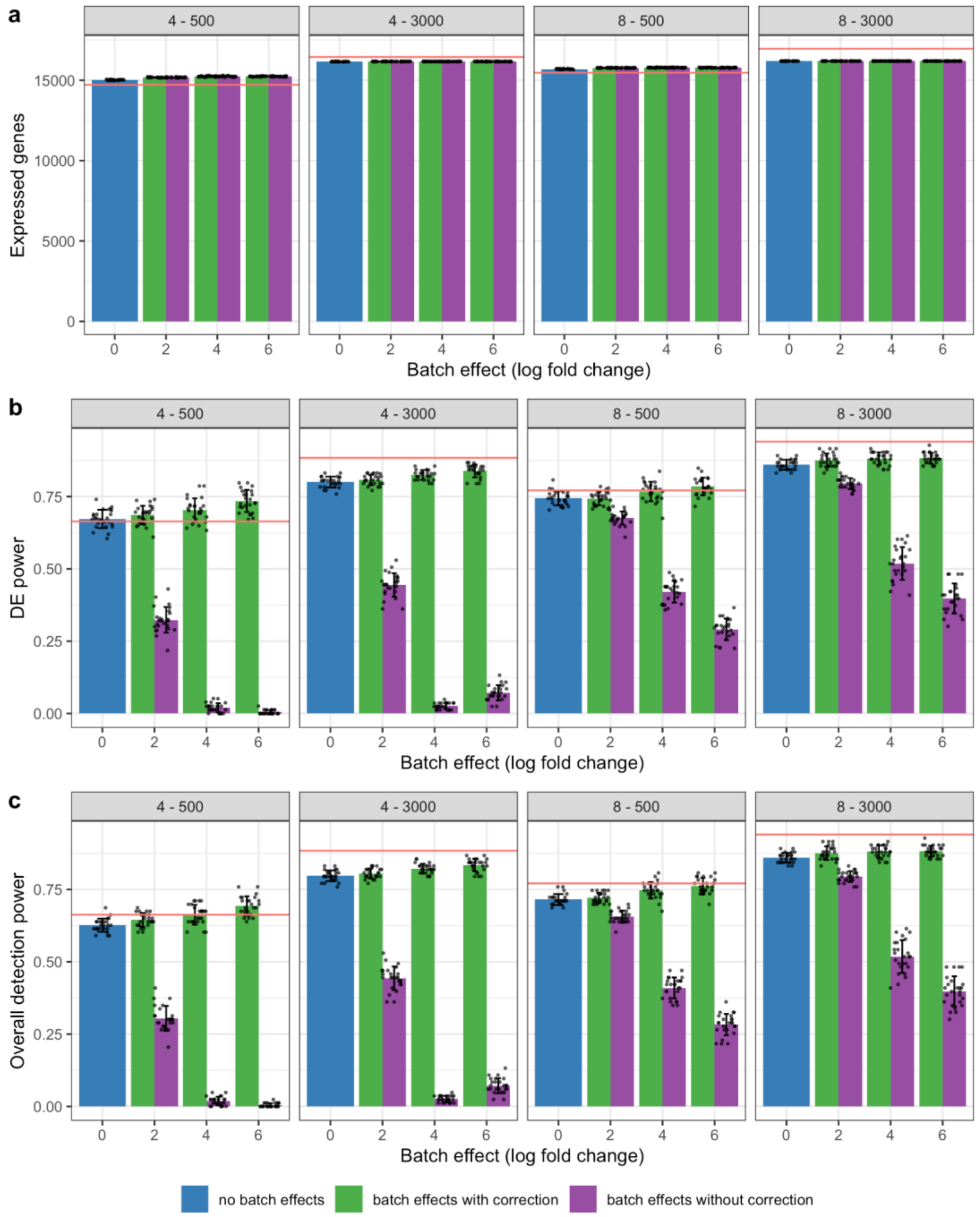

Figure S15: *Evaluating influence of batch effects on power.* We use powsimR for investigating the influence of batch effects on the number of expressed genes **(a)**, the DE power **(b)** and the overall detection power **(c)** based on simulated data with batch effects of different log fold change (x-axis). The effect was evaluated for different parameter combinations of sample size - number of cells per sample (see panel titles). The red lines visualize the prediction of scPower for this parameter combination. The power drops considerably if the log fold change of the batch effects is not corrected using covariates in the edgeR (violett), while with correction (green) it stays at the same level as without batch effects (blue). The barplots represent the mean power over n=25 simulation runs, the error bar shows the standard deviation and the points represent each individual simulation run. scPower as an analytic solution provides always the same result (so n=1 here).

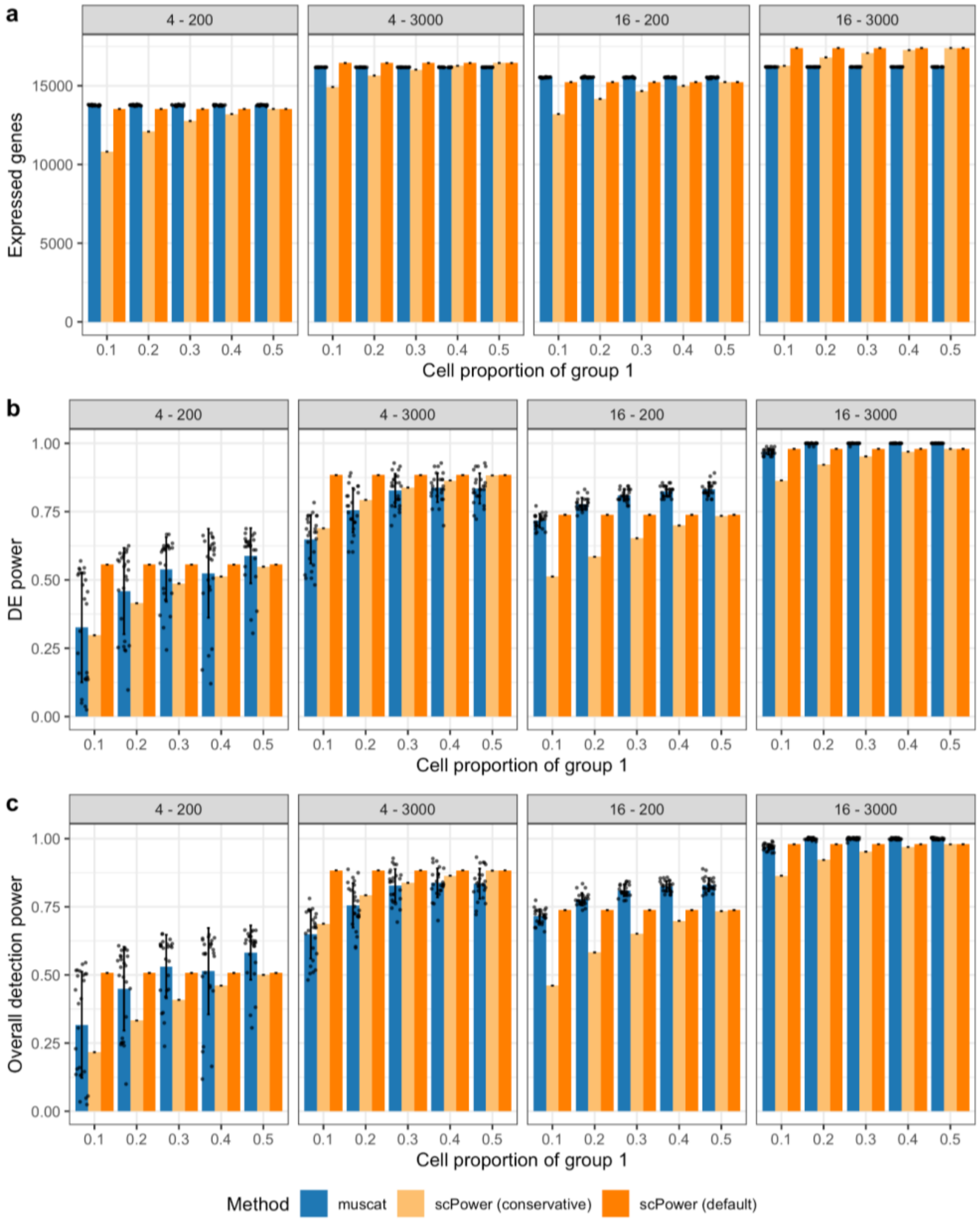

Figure S16: *Evaluating the influence of imbalanced cell proportions on the power.* We use muscat for investigating the influence of imbalanced cell proportions in both groups (x-axis) on the number of expressed genes (a), the DE power (b) and the overall detection power (c). The cell proportion here represents the fraction of cells that fall into group 1 from all measured cells of the cell type (e.g. a value of 0.3 means that 30% of measured cells belong to group 1 and 70% to group 2). The effect was evaluated for different parameter combinations of sample size - number of cells per sample (see panel titles). The power estimated with muscat (blue) is for small sample sizes lower than the default scPower estimation that assumes balanced groups with each the target cell type frequency (dark orange). However, a conservative estimation of scPower can be reached by scaling the cell type frequency by the cell proportion of group 1 (light orange). This represents a good lower bound power estimation for scenarios with imbalanced cell proportions between both groups. The barplots represent the mean power over n=25 simulation runs, the error bar shows the standard deviation and the points represent each individual simulation run. scPower as an analytic solution provides always the same result (so n=1 here).

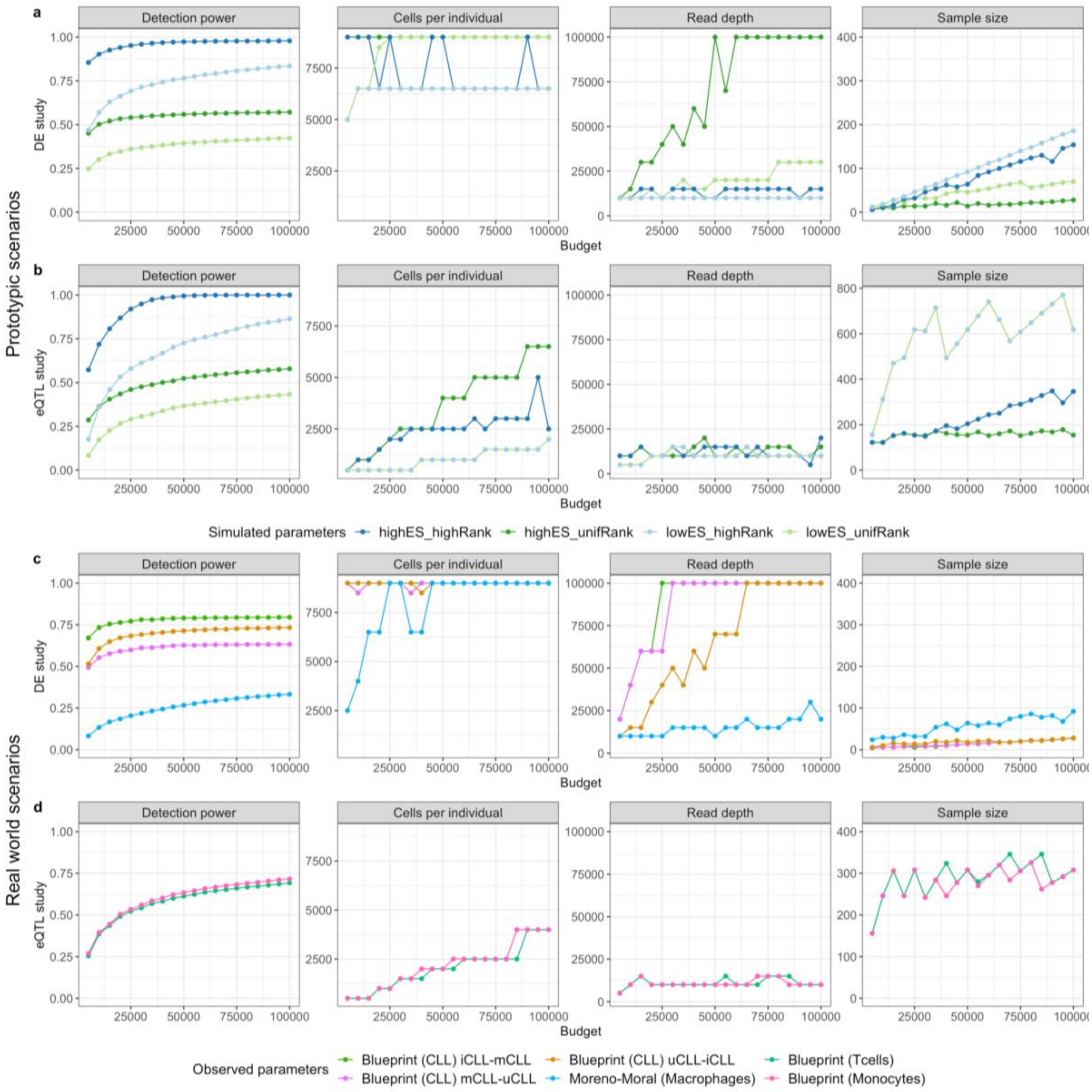

Figure S17: *Variant of Main Figure 5 created with different multiple testing correction - Optimal parameters for varying budgets and 10X Genomics data*. In contrast to Main Figure 5, FWER adjusted p-values were used for DE power. The figure shows the maximal reachable detection power (y-axis, first column) for a given experimental budget (x-axis) and the corresponding optimal parameter combinations for that budget (y-axis, second till fourth column). The colored lines indicate different effect sizes and gene expression rank distributions.

a,b Different simulated effect sizes and rank distributions (simulation names see text) for DEG studies **(a)** and eQTL studies **(b)** with models fitted on 10X PBMC data.

c,d Effect sizes and rank distributions observed in cell type sorted bulk RNA-seq DEG studies **(c)** and eQTL studies **(d)** with model fits analogously to **(a,b)**.

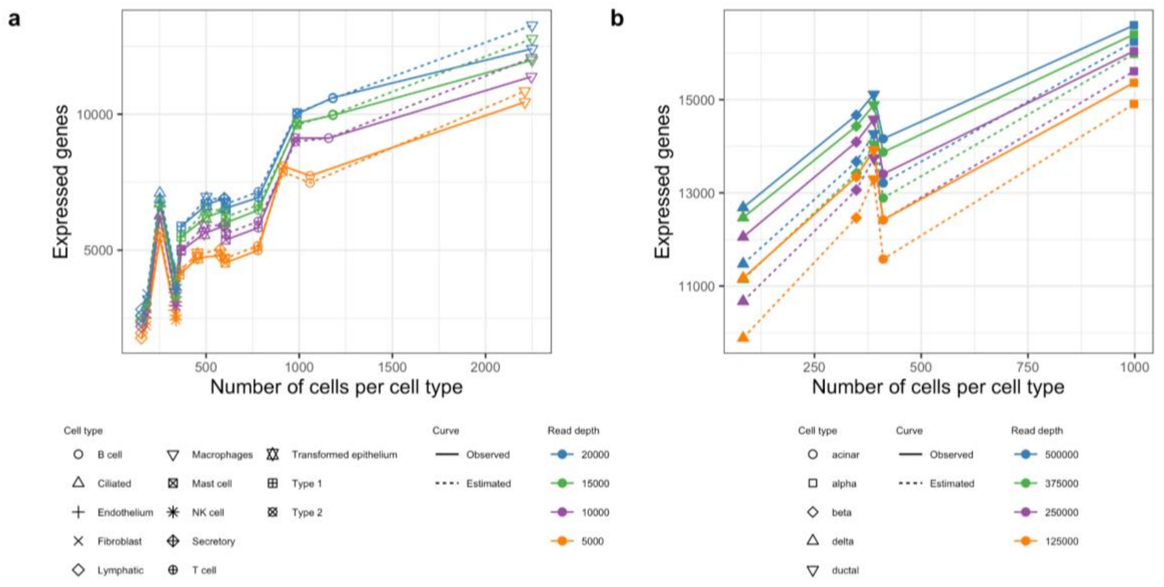

Figure S18: *Gene curve fits for different single cell technologies*. Evaluation of the expression probability from scPower for a lung data set measured with Drop-seq **(a)** and for a pancreas data set measured with Smart-seq2 **(b)**, both subsampled to different read depths (represented by line color). The solid lines represent the observed gene curves, the dashed lines the fitted curves. The point symbol visualizes the cell type. Gene expression criteria are chosen as UMI counts > 10 in all cells for Drop-seq **(a)** and read counts > 10 per kilobase transcript in all cells for Smart-Seq2 **(b)**.

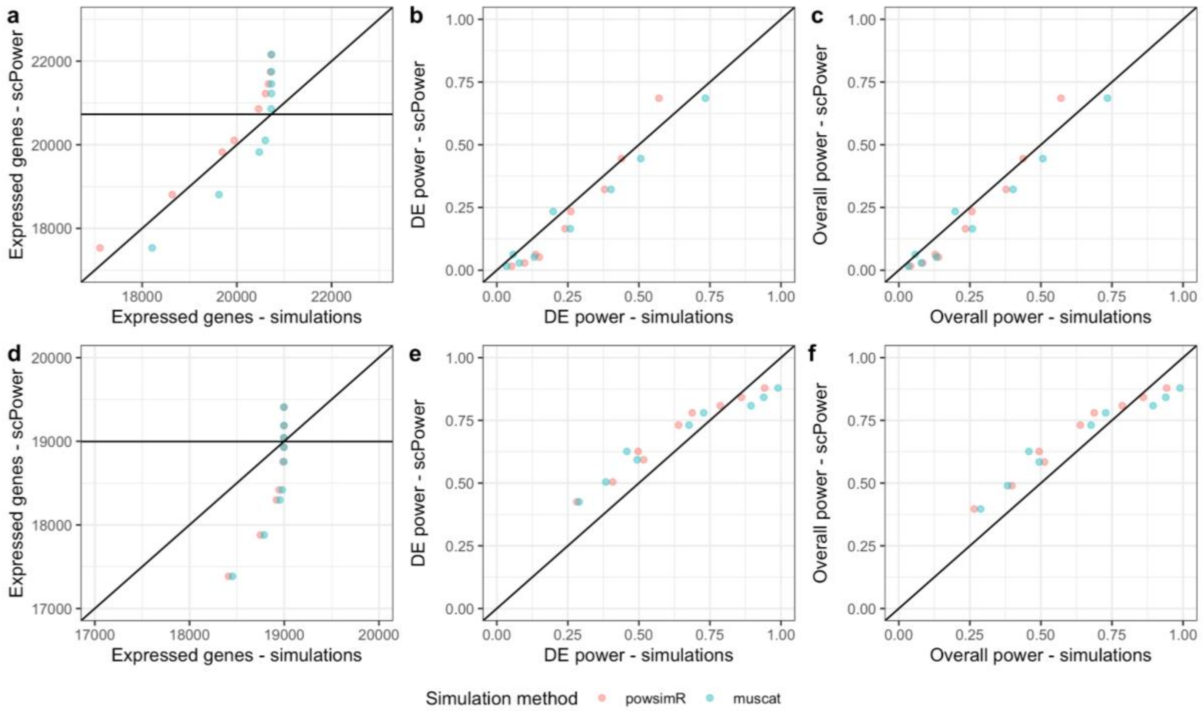

Figure S19: *Comparison of scPower with the simulation-based methods powsimR and muscat for Drop-Seq (a-c) and Smart-seq (d-f).* Difference in number of expressed genes **(a,d)**, DE power **(b,e)** and overall detection power **(c,f)** between scPower (y axis) and simulations of powsimR and muscat (x axis). The adapted version of powsimR (see methods) was run with edgeR-LRT using the mean-ratio method (MR) for normalization and the adapted version of muscat with edgeR. The power was evaluated for sample sizes of 4, 8 and 16 and for 200, 1000 and 3000 cells per person, always using FDR adjusted p-values.

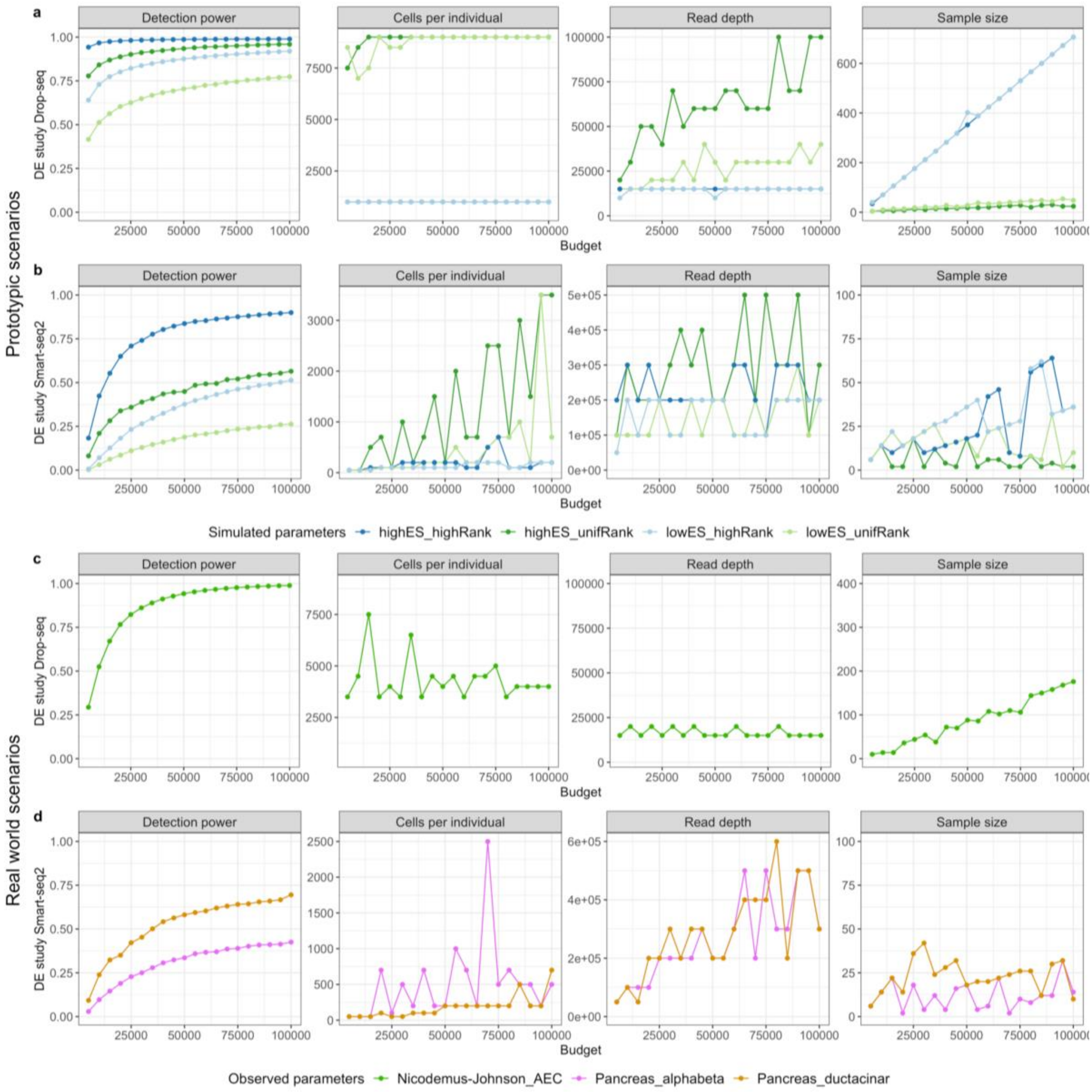

Figure S20: *Optimal parameters for varying budgets and Drop-seq and Smart-seq2 data*. The figure shows the maximal reachable detection power (y-axis, first column) for a given experimental budget (x-axis) and the corresponding optimal parameter combinations for that budget (y-axis, second till fourth column). The colored lines indicate different effect sizes and gene expression rank distributions.

a,b Different simulated effect sizes and rank distributions for DE studies with models fitted on Drop-seq lung data **(a)** and Smart-seq2 pancreas data **(b)**. highES = high effect sizes, lowES = low effect sizes, highRank = high expression ranks and unifRank = uniformly distributed expression ranks (always relative to effect sizes observed in published studies).

c,d Effect sizes and rank distributions observed in cell type sorted bulk RNA-seq DE studies with model fits analogously to **(a-b)**.

Supplementary Tables

	scPower	powsimR [40]	scDesign [42]	POWSC [43]	Muscat [41]	Mandric et al. [50]	SCOPIT [39]	How many cells [37]	SCEED [38]	Heimberg et al. [75]
DE power										
DE multi-sample power										
eQTL power										
Cell type detection power										
Transcriptional program power										
Simulation (orange) / Analytic (green)	A	S	S	S	S	A	A	A	S	A
Effect size based										
Optimize design										
Considering overloading of lanes										

Table S1: *Selected methods for power calculation in single cell RNA-seq.* The different methods (columns) cover different aspects of power calculation in single cell RNA-seq (row 1-5) and different approaches on how to assess the power (row 6-9).

Run	Donors	Target cell number	Target reads per cell	Estimated number of cells	Mean reads per cell
Run 1	1-14	8,000	50,000	7,491	40,650
Run 2	1-7	8,000	50,000	5,989	127,685
Run 3	8-14	8,000	50,000	8,144	13,949
Run 4	1-14	8,000	50,000	7,429	35,417
Run 5	1-14	8,000	50,000	7,765	21,057
Run 6	1-14	25,000	50,000	20,126	51,792

Table S2: *Training PBMC data set*. Experimental parameters of the 6 PBMC runs. In Run 1, 4, 5 and 6 all 14 donors were measured, in Run 2 only donor 1-7 and in Run 3 only donor 8-14. Run 6 was overloaded with 25,000 cells. The estimated number of cells and mean reads per cell are taken from the cell ranger summary statistics.

		UMI cutoff > 0		UMI cutoff > 10	
Run	Technology	r^2 value	p-value r	r^2 value	p-value r
PBMC1 - Run 1	10X Genomics	0.982	9.11e-21	0.997	1.12e-29
PBMC1 - Run 2	10X Genomics	0.990	2.01e-23	0.982	9.76e-21
PBMC1 - Run 3	10X Genomics	0.993	4.39e-25	0.997	5.46e-30
PBMC1 - Run 4	10X Genomics	0.994	1.17e-25	0.994	3.35e-26
PBMC1 - Run 5	10X Genomics	0.994	2.48e-26	0.997	9.12e-29
PBMC1 - Run 6	10X Genomics	0.993	1.03e-29	0.993	2.47e-29
PBMC2 - Run A	10X Genomics	0.873	2.03e- 3	0.925	5.41e- 4
PBMC2 - Run B	10X Genomics	0.981	1.74e- 5	0.994	1.09e- 6
PBMC2 - Run C	10X Genomics	0.942	2.81e- 4	0.992	2.25e- 6
PBMC2 - all runs	10X Genomics	0.934	1.20e-12	0.971	3.94e-16
Lung	Drop-seq	0.990	6.34e-52	0.995	9.83e-60
Pancreas	Smart-seq2	0.980	8.25e-17	0.991	4.61e-20

Table S3. *Evaluation of scPower gene expression prediction.* We compare the observed number of expressed genes and the number estimated by scPower for each data set (separated by batches) and for different UMI expression cutoffs (>0 or >10), corresponding to Figure 2B-C,S6,S7,S18. PBMC1 represents the training PBMC data set from (**Main Figure 3)** and PBMC2 the test PBMC data set. The difference is quantified using r^2 (Pearson correlation) and the Pearson correlation p-value based on a two-sided T test without multiple testing adjustment.

Technology (Tissue)	Cell type	Number cells	Mean UMI/read counts per cell	Number genes	Single cell counts		Pseudobulk counts	
					Δ AIC < 10	FDR(LRT) >= 0.05	Δ AIC < 10	FDR(LRT) >= 0.05
Smart-seq2 (Pancreas)	Alpha cells	998	1,061,059.9	17,715	69.4%	62.4%	99.4%	99.9%
	Ductal cells	389	1,226,815.0	16,108	67.9%	60.8%	98.5%	98.8%
	Beta cells	348	935,449.5	15,798	77.9%	71.5%	98.9%	100%
	Acinar cells	411	873,913.5	15,651	81.8%	76.1%	99.7%	100%
10X (PBMCs)	CD4 T cells	2,755	4,865.5	13,537	100%	100%	100%	100%
	CD14+ Monocytes	1,162	4,425.5	12,057	100%	100%	100%	100%
	CD8 T cells	859	3,760.1	11,242	100%	100%	100%	100%
	NK cells	650	3,025.1	10,859	100%	100%	100%	100%
	B cells	410	3,913.6	9,780	100%	100%	100%	100%
	FCGR3A+ Monocytes	173	6,994.1	9,297	100%	100%	99.7%	100%
Drop-seq (Lung)	Macrophages	2,250	1,366.1	17,151	100%	100%	99.8%	100%
	B cell	1,181	1,099.6	15,356	100%	100%	100%	100%
	Type 2	988	1,343.1	14,545	100%	100%	100%	100%
	Secretory	598	861.2	12,660	100%	100%	99.9%	100%
	Ciliated	252	1,914.6	12,653	100%	100%	99.5%	100%
	T cell	781	747.3	12,606	100%	100%	100%	100%
	Transformed epithelium	502	1,104.8	12,604	100%	100%	100%	100%
	Mast cell	606	948.1	12,251	100%	100%	100%	100%
	Type 1	367	1,012.4	11,337	100%	100%	100%	100%
	Endothelium	333	713.6	9,688	100%	100%	100%	100%

	NK cells	338	615.0	8,842	100%	100%	100%	100%

Table S4. Evaluation of negative binomial (NB) versus zero-inflated negative binomial (ZINB) distribution for modelling of single cell counts (columns 6-7) and pseudo bulk counts (columns 8-9). Each gene with at least a count of 3 was evaluated, using both the Akaike Information Criterion (AIC) and the likelihood ratio test (LRT). Δ AIC was calculated as AIC(NB) - AIC(ZINB), the threshold of 10 for Δ AIC to identify models with little support was chosen according to [1]. The p-values of the LRT were corrected for multiple testing using Benjamini Hochberg.

Technology	Library preparation costs per cell	Sequencing costs per 1 million reads
10X Genomics	0.05 € - 0.12€	3.42 €
Drop-Seq	0.09 €	3.42 €
Smart-Seq2	13.00 €	3.42 €

Table S5: *Experimental cost per technology*. Library preparation cost estimation (per cell) and sequencing cost estimation (per 1 million reads) for three of the most common single cell RNA-seq technologies in Euro (€). For 10X Genomics, the cost depends on the number of cells per lane, an overloading of each lane with 20,000 cells generates costs of 0.05€ per cell, a loading with 8,000 cells per lane costs of 0.12€ per cell.

Cell type	Markers
CD4 T cells	IL7R, CD3D
CD14+ Monocytes	CD14, LYZ
B cells	MS4A1, CD79A
CD8 T cells	CD8A, CD8B, CD3D
NK cells	GNLY, NKG7
FCGR3A+ Monocytes	FCGR3A, MS4A7
Dendritic cells	FCER1A, CST3
Megakaryocytes	PPBP
Plasma cells	CD79A

Table S6: *Marker genes*. Marker genes used to assign the Louvain clusters to the cell types. Annotations taken from van der Wijst et al., 2018 [2] and the Scanpy PBMC tutorial [3].

References

1. Burnham, K. P., Anderson, D. R. & Huyvaert, K. P. AIC model selection and multimodel inference in behavioral ecology: some background, observations, and comparisons. *Behav. Ecol. Sociobiol.* **65**, 23–35 (2011).
2. van der Wijst, M. G. P. *et al.* Single-cell RNA sequencing identifies celltype-specific cis-eQTLs and co-expression QTLs. *Nat. Genet.* **50**, 493–497 (2018).
3. Preprocessing and clustering 3k PBMCs — Scanpy documentation. https://scanpy-tutorials.readthedocs.io/en/latest/pbmc3k.html.
