# Supplementary material for: scPower accelerates and optimizes the design of multi-sample single cell transcriptomic studies
Source: Nat Commun. 2021 Nov 16;12:6625. doi: 10.1038/s41467-021-26779-7 (PMC8595682; doi:10.1038/s41467-021-26779-7)
Supplement: Supplementary file 3 — Reporting Summary [file 41467_2021_26779_MOESM3_ESM.pdf]

nature research

Corresponding author(s): Matthias Heinig

Last updated by author(s): Oct, 15, 2021

Reporting Summary

Nature Research wishes to improve the reproducibility of the work that we publish. This form provides structure for consistency and transparency in reporting. For further information on Nature Research policies, see our Editorial Policies and the Editorial Policy Checklist.

Statistics

For all statistical analyses, confirm that the following items are present in the figure legend, table legend, main text, or Methods section.

n/a	Confirmed	
☐	☒	The exact sample size (*n*) for each experimental group/condition, given as a discrete number and unit of measurement
☐	☒	A statement on whether measurements were taken from distinct samples or whether the same sample was measured repeatedly
☐	☒	The statistical test(s) used AND whether they are one- or two-sided<br>*Only common tests should be described solely by name; describe more complex techniques in the Methods section.*
☐	☒	A description of all covariates tested
☐	☒	A description of any assumptions or corrections, such as tests of normality and adjustment for multiple comparisons
☐	☒	A full description of the statistical parameters including central tendency (e.g. means) or other basic estimates (e.g. regression coefficient) AND variation (e.g. standard deviation) or associated estimates of uncertainty (e.g. confidence intervals)
☐	☒	For null hypothesis testing, the test statistic (e.g. *F*, *t*, *r*) with confidence intervals, effect sizes, degrees of freedom and *P* value noted<br>*Give P values as exact values whenever suitable.*
☒	☐	For Bayesian analysis, information on the choice of priors and Markov chain Monte Carlo settings
☒	☐	For hierarchical and complex designs, identification of the appropriate level for tests and full reporting of outcomes
☐	☒	Estimates of effect sizes (e.g. Cohen's *d*, Pearson's *r*), indicating how they were calculated

Our web collection on statistics for biologists contains articles on many of the points above.

Software and code

Policy information about availability of computer code

Data collection	The PBMC data set was mapped using cellranger (v2.0.0 and v2.1.1), doublets detected using Demuxlet (v1.0) and Scrublet (v0.1). Basic filtering of cells was performed using Scanpy (v1.4). Reads were subsampled using fastq-tools (v0.8).
Data analysis	The main code is available through https://github.com/heiniglab/scPower and based on open source R packages of MKmisc (v1.6), pwr (v1.3-0) and DESeq (v1.34.1). For evaluation of the methods, the R packages muscat (v1.4.0) and powsimR (v1.2.3) in combination with DESeq2 (v1.22.2), edgeR (3.32.1) and limma (3.46.0) were used.

For manuscripts utilizing custom algorithms or software that are central to the research but not yet described in published literature, software must be made available to editors and reviewers. We strongly encourage code deposition in a community repository (e.g. GitHub). See the Nature Research guidelines for submitting code & software for further information.

Data

Policy information about availability of data

All manuscripts must include a data availability statement. This statement should provide the following information, where applicable:

- Accession codes, unique identifiers, or web links for publicly available datasets
- A list of figures that have associated raw data
- A description of any restrictions on data availability

The single cell PBMC data set generated and analysed during the current study is available on Gene Expression Omnibus (GEO) with accession number GSE185714 [https://www.ncbi.nlm.nih.gov/geo/query/acc.cgi?acc=GSE185714].
The other single cell test data sets are available on GEO with accession numbers GSE96583 [https://www.ncbi.nlm.nih.gov/geo/query/acc.cgi?acc=GSE96583], GSE130148 [https://www.ncbi.nlm.nih.gov/geo/query/acc.cgi?acc=GSE130148] and GSE81547 [https://www.ncbi.nlm.nih.gov/geo/query/acc.cgi?acc=GSE81547].
The effect sizes for the eQTL and DE power were taken from published studies, accessible in the supplement of Chen et al, 2016 (doi: 10.1016/j.cell.2016.10.026),

Rendeiro et al, 2016 (doi: 10.1038/ncomms11938), Moreno-Moral et al, 2018 (doi: 10.1136/annrheumdis-2017-212454) and Arda et al, 2016 (doi: 10.1016/j.cmet.2016.04.002). For one data set, we reanalysed the count matrix at GEO with accession number GSE85567 [https://www.ncbi.nlm.nih.gov/geo/query/acc.cgi?acc=GSE85567] to get the effect sizes.

Field-specific reporting

Please select the one below that is the best fit for your research. If you are not sure, read the appropriate sections before making your selection.

- [x] Life sciences
- [ ] Behavioural & social sciences
- [ ] Ecological, evolutionary & environmental sciences

For a reference copy of the document with all sections, see nature.com/documents/nr-reporting-summary-flat.pdf

Life sciences study design

All studies must disclose on these points even when the disclosure is negative.

Sample size

The PBMC data set is a pilot experiment for a larger population study and all available samples were taken.
For the public available data sets, all available samples were taken.

Data exclusions

For the PBMC data set: no complete samples were removed, only cells within each sample. The removal of cells was performed according to established standards for single cell analysis (see Lueken et al, 2019): cells were removed that were identified as doublets/ambiguous by Demuxlet or Scrublet, had a high fraction of mitochondrial gene counts (>= 10%) or were outlier in their general count distribution (< 200 genes or >2500 genes).
For the publicly available data sets: no further data exclusion was performed, but the complete available and already prefiltered public data sets were used.

Replication

We report on an observational study, therefore no experimental groups were assigned and randomization was not relevant.

Randomization

We used all samples in all of the data sets, therefore randomization is not relevant in our study.

Blinding

The used samples had no disease status, but were all in the control group of the BeCOME study. Therefore blinding was not relevant.

Reporting for specific materials, systems and methods

We require information from authors about some types of materials, experimental systems and methods used in many studies. Here, indicate whether each material, system or method listed is relevant to your study. If you are not sure if a list item applies to your research, read the appropriate section before selecting a response.

Materials & experimental systems

n/a	Involved in the study	
x		Antibodies
x		Eukaryotic cell lines
x		Palaeontology and archaeology
x		Animals and other organisms
	x	Human research participants
	x	Clinical data
x		Dual use research of concern

Methods

n/a	Involved in the study	
x		ChIP-seq
x		Flow cytometry
x		MRI-based neuroimaging

Human research participants

Policy information about studies involving human research participants

Population characteristics

Ages of individuals were between 18 and 75 years. For this study, we selected 7 males and 7 females.

Recruitment

Control individuals were recruited at the Max Planck Institute of Psychiatry (MPIP) in Munich, Germany as part of the BeCOME cohort, an observational and exploratory study which combines deep phenotyping and omics data in order to gain a better understanding of the biological basis of mental disorders.

Ethics oversight

The study was approved by the local Ethics Committee of the Ludwig Maximilians University, Munich, Germany, and written informed consent is obtained from all participants. Control individuals agreed to donate PBMCs for potential use with high-throughput sequencing.

Note that full information on the approval of the study protocol must also be provided in the manuscript.

Clinical data

Policy information about clinical studies

All manuscripts should comply with the ICMJEguidelines for publication of clinical research and a completedCONSORT checklist must be included with all submissions.

Clinical trial registration	NCT03984084
Study protocol	Full protocol is described in Brueckl et al., BMC Psychiatry 2020, doi: 10.1186/s12888-020-02541-z
Data collection	Individuals were recruited through an ongoing collection that began in 2015. Multiple biological and clinical levels were assessed in both patients and controls, as described in detail in Brueckl et al.
Outcomes	Psychiatric control status was determined using a fully structured diagnostic interview (DIAX/M-CIDI). Other exclusion criteria included substance abuse, metal retardation, myopia, as well as pregnancy or postpartum.
